# Low back pain and its determinants among wait staff in Gondar town, North West Ethiopia: A cross-sectional study

**DOI:** 10.3389/fpain.2022.964297

**Published:** 2022-09-06

**Authors:** Ermias Solomon Yalew, Kedir Sany Adem, Alemu Kassaw Kibret, Moges Gashaw

**Affiliations:** Department of Physiotherapy, School of Medicine, College of Medicine and Health Sciences, University of Gondar Comprehensive Specialized Hospital, Gondar, Ethiopia

**Keywords:** low back pain, wait staff, waiters, waitress, Gondar, Ethiopia

## Abstract

**Background:**

Low back pain is a common public health issue in the working population and one of the leading causes of disability. It is the leading cause of work-related conditions and the most common reason for filing a workers' compensation claim in low- and middle-income countries. Ethiopia is a developing country; there is a shortage of working materials, skilled labor, and a lack of awareness of ergonomics posture, which lead to lifting heavy objects, long periods of standing, repetitive twisting, and same sustained posture for long periods of time without a shift. As a result, the purpose of this study was to assess the prevalence and associated factors of work-related low back pain among restaurant wait staff in Gondar, Ethiopia, in the year 2019.

**Methods:**

Institution-based cross-sectional study, including 420 restaurant wait personnel, was undertaken from 1 March to 30 April 2019. A simple random sampling procedure was used to choose the restaurants and wait staff. A standardized Nordic questionnaire was used to collect data. Data were entered into Epi Info 7 and analyzed in SPSS version 20. The univariate and multivariate logistic regression analyses were calculated. The significance of associations was reported by a *P*-value of < 0.05 and an adjusted odds ratio (AOR). The model fitness checked by the Hosmer–Lemeshow goodness of fit test was used.

**Result:**

In this study, a total of 420 participants (99.53% response rate) ranging in age from 17 to 53 years old participated, with 184 (43.8%) participants reporting low back pain at some point in the past 12 months. Female participants had a higher prevalence of 130 (70.6%). Sex (AOR = 2.98; 95% CI: 1.07–8.30), frequent exercise (AOR 0.47; 95% CI: 0.24, 0.93), extended standing (AOR 8.82; 95% CI: 3.30, 20.32], and repetitive tasks (AOR 7.49; 95% CI: 4.29, 13.19) were all found to be significant predictors in low back pain.

**Conclusion:**

More than two-fifth of waitresses and waiters reported low back discomfort at some point in the past 12 months. Predisposing factors for low back discomfort among restaurant wait staff included being female, standing for long periods of time while serving, and performing repetitive tasks. Regular exercise was found to be a protective factor against low back pain in wait staff. Delivering ongoing safety training is among the most potent essential measures required in preventing low back pain.

## Introduction

Musculoskeletal injuries are a broad term that refers to a variety of inflammatory, degenerative diseases, and disorders that cause pain and functional impairments in people who are exposed to work activities and conditions that contributed to the development or exacerbation of the condition but did not act as the sole cause (1). Despite the identification of several associated factors (such as work posture, long periods of standing, moving heavy objects, repetitive twisting forward and backward, obesity, and aging), the reasons for low back pain remain unknown, making diagnosis challenging (2).

According to the WHO, 50–70% of workers suffer from work-related musculoskeletal disorders (WMSDs). WMSDs afflict around 317 million people each year, with 6,300 people dying every day (3). According to the United States Bureau of Labor Statistics, back injuries account for 20% of all workplace injuries and illnesses and nearly 25% of annual workers' compensation payouts. Based on a recent assessment by the United States National Safety Council, overexertion is the most common cause of occupational injury, accounting for 31% of all injuries (4). In many parts of the world, low back pain is the leading cause of activity limitation and work absence, imposing a substantial financial load on individuals, families, and governments (5).

Low back pain is described as “pain and discomfort, situated below the costal edge and above the inferior gluteal folds, with or without leg pain,” according to European standards for preventing low back pain (6). Low back pain (LBP) is a common health concern among the general public, and it is one of the leading causes of disability, negatively impacting work performance and wellbeing. Work-related low back pain (WLBP) is a musculoskeletal condition that is described as any back pain thought to be induced by occupational exposures. This illness is also known as overuse syndrome, repetitive strain injury, or cumulative trauma disorder (7). WLBP is a type of low back pain that occurs due to work and is clinically determined to have been caused, at least in part, or exacerbated by the work environment (8).

In affluent countries, a variety of initiatives have been implemented to mitigate the impact. As a result, the severity and cost of lower back pain are decreasing, absenteeism from work and medical costs are falling, working conditions are improving, and many factors that lead to the development of lower back pain are being discovered (8). However, the burden of low back pain was exacerbated in developing countries because the types of work, working conditions, and other factors contributing to the development of lower back pain among different working groups, including restaurant wait staff, were unknown (9).

In Ethiopia, the tourism industry is occasionally booming and hiring a large number of people in the hotel and other sectors. However, the working environment is hazardous to the worker, and health and safety systems are inadequately implemented. Furthermore, most working materials and skilled manpower are insufficient, thus the behavior of work in wait staff requires lifting heavy objects, long periods of standing, repetitive twisting, and the same sustained posture for long periods of time without a shift. These can be the leading causes of low back pain in Ethiopia, and there was a lack of information on the prevalence and associated factors of low back pain among waiters and waitresses in Ethiopia, particularly in Gondar town. As a result, the purpose of this study was to determine the prevalence and associated factors of low back pain.

## Materials and methods

### Study design, area, and period

A cross-sectional study was conducted among restaurant wait staff in Gondar, Ethiopia. The research was carried out from 1 March to 30 April 2019 in Gondar, a town in northern Ethiopia. It is located 750 km from Addis Ababa, Ethiopia's capital city. In Gondar, there are 101 restaurants, and 1,309 restaurant workers serve customers in food preparation, cooking, distribution, food hygiene, service cleaning, and cashier positions.

### Source population and study population

All the restaurant wait staff working at restaurants (hotels) and the selected restaurants in Gondar town were the source and study population of this study, respectively.

### Inclusion and exclusion criteria

All the restaurant wait staff working at Gondar town restaurants for at least 12 months were included in this study, whereas restaurant wait staff with physical deformities (such as excessive lumbar lordosis, increased thoracic kyphosis, and scoliosis) (10), a history of traumatic low back pain, back surgery, or medically diagnosed low back pain were excluded.

### Sample size determination

The sample was determined by using a single population proportion formula on the following assumption (11). Level of significance (α): 5% (with a confidence level of 95%), marginal error: 5% P: is the prevalence of low back pain among waiters that is 50% because no studies were conducted in this area in our country.

The Z-value of 1.96 was used at 95% CI (n: sample size, P: proportion, d: marginal error).


$$\begin{array}{c} \frac{{\left(Z_{a/2}\right)}^{2}\;*\;P(1-P)}{d^{2}}\\ \frac{{\left(1.96\right)}^{2}\;*\;0.5(0.5)}{0.05^{2}}\\ n=384 \end{array}$$

The total sample size (n) with a 10% nonresponse rate becomes 422.

### Sampling procedure

A simple random sampling was used to select the study subjects. The study participants were selected from 40 restaurants in Gondar town. Each restaurant consisted of an average of 13 waiting staff. To ensure representativeness, first, a proportional allocation of the participants was done for each restaurant, and then waiters and waitresses from those restaurants were selected using a simple random sampling approach.

### Operational definition

#### Body mass index

Weight in kilogram divided by the square of the height in meters (kg/m^2^); **underweight** < 18.50 kg/m^2^, **normal** 18.50–24.99 kg/m^2^, and **overweight** ≥ 25 kg/m^2^ (12).

#### Low back pain

A pain and discomfort, localized below the costal margin and above the inferior gluteal folds, with or without leg pain (13).

#### Nonspecific low back pain

A type of low back pain not attributed to recognizable, known specific pathology (14).

#### Repetitive task

Workers put to repetitive tasks that recur every 30 s in the same direction in < 30 s (15).

#### Regular physical exercise

Performing any type of physical exercise for 30 min at least two times each week (8).

#### Prolonged standing

Standing for more than 4 h (16).

### Data collection instrument

Face-to-face interviews were used to gather information. The study participants' low back pain was assessed using the standardized Nordic questionnaire for the evaluation of musculoskeletal symptoms. The questionnaire was designed to determine the prevalence of musculoskeletal issues in a certain population while also considering where they occur in the body (17). The questionnaire had four components, which are sociodemographic, personal and psychological, occupational and ergonomic, and low back pain-related questions (Supplementary File I).

### Data quality control

The questionnaire was written in English, translated into Amharic, and then back into English by language experts. The questionnaire's Amharic translation was pretested in Bahir Dar town's eateries with 5% of the total sample size and required corrections were made based on the results. Three data collectors were in charge of data collection. The principal investigator (ES) provided the data collectors with a 2-day comprehensive training on how to approach study participants, how to use the questionnaire and guidelines, and data collection procedures. The investigators kept a close eye on the data collection technique and evaluated the obtained questionnaire on a regular basis for accuracy, completeness, and consistency.

### Data management and analysis

The obtained data were coded and reviewed for completeness, missing values, and clarity by the primary investigator and supervisor at the time of data entry. The Epi Info 7 was used to enter the coded data, which was then exported, processed, and analyzed using SPSS version 20. Frequency, mean, SD, and tables were used to present the findings of descriptive statistics. Binary logistics regression was conducted to identify the associations between dependent and independent variables. Independent variables with *p*-values of 0.2 in the univariate analysis were taken to the multivariate logistic regression analysis to control the effects of potential confounders.

A *p*-value of 0.05 (95% CI) and an adjusted odds ratio (AOR) were used to determine the significance of the associations. The model fitness was checked by the Hosmer–Lemeshow goodness of fit test, with a *p*-value > 0.05.

## Results

### Sociodemographic characteristics of study participants

A total of 420 participants aged 17–53 years participated in this study. This is a 99.53% response rate and is beyond the power calculated sample size (*n* = 384).

Out of the 420 respondents interviewed, 257 (61.2%) of the participants were female participants. Two-thirds (63.3%) of the participants were aged 17–24 years. The mean (SD) year of experience of the waiters was 1.9 (0.6) years. Two-thirds (65%) of the respondents had work experience of 2–5 years. Three-fourths (70.7%) of participants had part-time jobs in addition to their waiting jobs. More than half (63.3%) of the participants' work conditions were during the daytime (Table 1).

**Table 1:** Socio-demographic characteristics of restaurant wait staff in Gondar town, Ethiopia, 2019 (*n* = 420).

**Variable**	**Category**	**Frequency (n)**	**Percent (%)**
Age	17–24	265	63.1
	25–34	137	32.6
	35–53	18	4.27
Sex	Female	257	61.2
	Male	163	38.8
Marital status	Currently unmarried	298	71.0
	Currently married	122	29.0
Religion	Orthodox	359	85.5
	Protestant	29	6.9
	Muslim	24	5.7
	Catholic	7	1.7
Education level	Can't read and write	8	1.9
	Can read and write	22	5.2
	Primary school	82	19.5
	Secondary school	220	52.4
	Collage and above	88	21.0
Work condition status	Day	266	63.3
	With shift day and night	137	32.6
	Night	17	4.1
Year of experience	0–1	102	24.3
	2–5	273	65.0
	6–10	40	9.5
	≥11	5	1.2
Additional job	Yes	297	70.7
	No	123	29.3

### Individual and behavioral characteristics of the participants

Out of 420 respondents interviewed, 200 (47.6%) participants had a BMI of 18.50–24.99 kg/m^2^. More than two-fifths (43.1%) of the participants took ergonomic training, one-third (28.1%) of the participants had knowledge about lower back ergonomics, and more than one-third (39.7%) of the participants never had regular exercise before. Three-fourths (74.7%) of the participants were satisfied with their comfortable daily activity. A total of 132 (71.7%) waiters felt happy at work, but 117 (27.9) waiters were bothered by feeling senseless and under little pressure due to their work, and also 259 (61.7%) waiters felt fatigued due to their workload (Table 2).

**Table 2:** Personal and psychological characteristics of restaurant wait staff in Gondar town, Ethiopia, 2019 (*n* = 420).

**Variables**	**Category**	**Frequency (*n*)**	**Percent (%)**
BMI	<18.50	155	37
	18.50–24.99	200	47.6
	>25	65	15.4
Ergonomic training	Yes	181	43.1
	No	239	56.9
Knowledge of back ergonomics	Yes	118	28.1
	No	302	71.9
Habit of doing regular exercise	Never exercise	166	39.5
	Sometimes	180	42.9
	Usually	74	17.6
Are you satisfied for being waiter	Yes	314	74.8
	No	106	25.2
Comfortable with daily activity	Yes	334	79.5
	No	86	20.5
Mental stressed being waiter	Yes	142	33.8
	No	278	66.2
Sleep disturbance	Yes	99	23.6
	No	321	76.4
Feeling senseless and little pleasure	Yes	117	27.9
	No	303	72.1
Fatigue because of daily workload during	Yes	259	61.7
	No	161	38.3
Satisfied with income	Yes	179	42.6
	No	241	57.4
Satisfied with work	Very dissatisfied	158	37.6
	Dissatisfied	80	19
	Neutral	51	12.1
	satisfied	122	29.0
	Very satisfied	9	2.1

### Occupational and ergonomics factors of the restaurant wait staff

Out of 420 participants, 84.5% of the participants felt LBP while bending or twisting. Nearly, three-fourths of the participants (69.3%) complain about LPB during standing. Almost all of the participants (92.4%) did not complain about LBP during sitting position (Table 3).

**Table 3:** Occupational and ergonomics factors of restaurant wait staff in Gondar town, Ethiopia, 2019 (*n* = 420).

**Variable**	**Category**	**Frequency (*n*)**	**Percent (%)**
Bending / twisting	Yes	356	84.8
	No	64	15.2
Lifting	Yes	72	17.1
	No	348	82.9
Standing	Yes	291	69.3
	No	129	30.7
Sitting	Yes	32	7.6
	No	388	92.4
Forming repetitive tasks	Yes	115	27.4
	No	305	72.6
Working in an awkward / cramped position	Yes	25	6
	No	395	94
Working when physically fatigued	Yes	23	5.5
	No	397	94.5
Feel pain on your low back more at night shift different from the day	Yes	77	18.3
	No	343	81.7

### Low back pain prevalence among restaurant wait staff

Of 420 respondents, 184 (43.8%) respondents experienced low back pain throughout their job careers. Of the respondents with LBP in the last 6 months, 52 (12.4%) respondents were absent from their work due to LBP. In this study, the prevalence of LBP was higher among female waiters (70.6%) than among male waiters [54 (29.3%)]. Among the BMI group of waiters, a higher prevalence of LBP was observed in the lowest BMI groups (<25) of waiters. It is also higher among waiters who had sleeping disturbance [127 (69.1%)] than those who had no sleeping disturbance [57 (30.9%)].

### Factors associated with low back pain

In the multivariate logistic regression analysis, sex, regular exercise, prolonged standing, and repetitive tasks were variables that were significantly associated with low back pain among restaurant wait staff.

Female restaurant wait staff had 2.98 times more low back pain than male wait staff [adjusted odds ratio (AOR): 2.98 (1.07–8.30)]. Restaurant wait staff who exercised on a regular basis was 53% less likely to have low back pain than restaurant wait staff who did not do exercise on a regular basis [AOR): 0.47 (0.24–0.93)]. The odds of having low back pain were 8.82 times higher for restaurant wait staff who had prolonged standing than for restaurant wait staff who did not have prolonged standing [AOR: 8.82 (3.30–20.32)]. Restaurant wait staff who had a repetitive task had 7.49 more low back pain than restaurant wait staff who did not have a repetitive task [AOR: 7.49 (4.29–13.19)] (Table 4).

**Table 4:** Bi-variable and multivariable logistic regression analysis on factors associated with low back pain among restaurant wait staff in Gondar town, Ethiopia, 2019 (*n* = 420).

**Variables**	**LBP**			**Crude OR (95% CI)**	**Adjusted OR (95% CI)**	***p*-value**
		**Yes**	**No**			
Sex	Female	130 (50.6%)	127 (49.4%)	3.15 (2.04–4.85)	2.98 (1.07–8.30)	0.04*
	Male	40 (24.5%)	123 (75.5%)	1	1	
Marital status	Not Currently married	107 (35.9%)	191 (64.1%)	1	1	
	Currently married	63 (51.6%)	59 (48.4%)	1.90 (1.24–2.91)	1.11 (0.65–1.87)	0.70
Additional part of job	Yes	124 (67.4%)	173 (73.3)	0.75 (0.49,1.15)	0.90 (0.42–1.94)	0.79
	No	60 (32.6%)	63 (26.7)	1	1	
Regular exercise	Never exercise	70 (42.2%)	96 (57.8%)	1	1	
	Sometimes	60 (33.3%)	120 (66.7%)	0.69 (0.44–1.06)	0.62 (0.31–1.27)	0.19
	Usually	40 (54.1%)	34 (45.9%)	1.61 (0.93–2.8)	0.47 (0.24–0.93)	0.03*
Prolonged standing	Yes	149 (51.2%)	142 (48.8%)	5.39 (3.20–9.08)	8.82 (3.30–20.32)	0.00*
	No	21 (16.3%)	108 (83.7%)	1	1	
Forming repetitive tasks	Yes	87 (75.7%)	28 (24.3%)	4.77 (3.07–7.40)	7.49 (4.29–13.19)	0.00*
	No	83 (27.2%)	222 (72.8%)	1	1	

1, reference category; AOR, Adjusted odds ratio; CI, confidence interval; COR, crudes odds ratio;

^*^statistically significant at p < 0.05.

## Discussion

The purpose of this study was to determine the prevalence of low back pain and its associated factors among restaurant wait staff in Gondar, Ethiopia. The overall prevalence of low back pain among restaurant wait staff was 43.8%, and variables such as sex, regular exercise, prolonged standing, and repetitive tasks were significantly associated with low back pain among restaurant wait staff.

The magnitude of work-related low back pain in this study is lower than in research, which was conducted in Taiwan (52.7%) (18). This difference observed in the prevalence rate of LBP could be due to the difference in the study setting, sample size, and the study participant's characteristics. The Taiwan study was conducted among 905 restaurants and hotel workers with a large sample size when compared to the present study. Another possible reason might be the difference in the study characteristics between the study participants. The Taiwan study assesses the work-related LBP pain with their pain intensity, while the present study did not assess the pain intensity and excluded the study participants who presented a previous history of LBP. In addition, a study done in Iran among steel workers found that 63.81% had experienced LBP (19). The main difference is that this study is conducted on restaurant workers, while the Iranian study was conducted among steel construction workers, which need high force and different ergonomic postures. The job of the wait staff is also manual. However, maybe a more plausible explanation could be the relatively higher intensity/level of manual work is higher among the steel industry workers. Furthermore, the study done in Ethiopia among teachers found that 57.5% had low back pain (16). This variation could be attributed to the sample size and the population studied in the preceding study, which included teachers suffering from low back pain. Similarly, in the study done in Ethiopia, Gondar, work-related low back pain among low-wage workers was 58.1% (10). The possible reason for this variation could be the variation in work nature, working time, and level of understanding of the ergonomics position. Teachers most commonly work in a standing position, while wait staff uses their back during bending and lifting.

In contrast, the prevalence of work-related low back pain among restaurant wait staff is higher than in studies conducted in the United States, at 18% among restaurant wait staff (20). The possible explanation for the variation in the current study may be that there is low access to information about occupational health and safety practices (21). Furthermore, this study's results are much higher than the studies done on first-class restaurant workers in Turkey (26%) (22). This variation might be due to the difference in the study participant and ways of the assessment procedure. The Turkey study was conducted in the selective study population with pain intensity and pain coping mechanism assessment among the first class wait staff, but our study was conducted among the whole wait staff which is not categorized by classes and underground mine workers in Zambia (24%) (23). The possible explanation for variation could be the difference in the sample size and sampling method. The Zambian study was conducted among 202 mining workers recruited with a stratified sampling technique, while this study was conducted with a large sample increased by double among wait staff with a simple random technique.

The findings of this research revealed that sex is significantly associated with work-related low back pain, which means a female is 3 times [AOR (1.07–8.30)] more likely to have work-related low back pain than compared to a male. This result was in line with the study, which was conducted in Iran (24), a literature review done in (25), and a systematic review done in Africa (26), Gondar, Ethiopia (16). One possible explanation is that women are more obese than men, which cause low back pain. Another possible explanation is that men exercise more frequently than women. Furthermore, women have a lower pain tolerance than men, and they are more likely to report any pain condition. Osteoporosis, menstruation, pregnancy, and childbirth may all play a role in the increased occurrence of LBP in women (16, 27).

The other variable that was significantly associated with work-related low back pain was regular physical exercise. Waiters/waitresses who exercised on a regular basis were 53% [AOR (0.24–0.93)] less likely to develop LBP than those who did not do regular exercise. This is similar to the research conducted in the United States (28), a systematic review done on leisure time physical activity and low back pain (29), Addis Ababa, Ethiopia (8), Gondar, Ethiopia (16). The possible explanation might be that shortened and weak muscles can cause LBP as they can cause misalignment of the spine. Exercises can strengthen, lengthen, and make the muscles of the back strong to support and keep the spine in perfect alignment for proper functioning (30).

Regarding prolonged standing, it was one of the associated factors of low back pain. In our research, it was about 9 times [AOR (3.30–20.32)] more likely to develop low back pain than not standing for a prolonged time. This is in line with the study conducted in Ethiopia, Addis Ababa (8), and Gondar, Ethiopia (16). Standing for extended periods of time places an undue strain on the lumbar spine and other anatomical systems, which can result in LBP.

In our study, performing repetitive tasks was one of the associated factors with low back pain in our study, and it was 7 times [AOR (4.29–13.19)] more likely to cause low back pain than not doing repetitive tasks. This result is the same as the study done in Taiwan (31). The repetition of identical motions, but also the repetition of multiple activities with motions that are quite similar utilize the same muscles and tissues. As a result, joints and muscles are vulnerable to repetitive motion injuries, and muscles may not have enough time to recover from the strain before the motion is repeated. There is additional data that show a strong link between repeated work and lower back pain (8). The organization should facilitate the wait staff's frequent resting and create an environment for regular exercise. The wait staff should avoid prolonged standing and practice ergonomic health and safety procedure to prevent work-related low back pain.

## Conclusion

More than two-fifths of waitresses and waiters reported low back discomfort at some point within 12 months. Waitresses with low back discomfort were more likely to be female, stand for lengthy periods of time while serving, and do repetitive tasks. Regular exercise was found to be a protective factor against low back pain in restaurant waiter employees. It is preferable to provide waiters/waitresses with ergonomic training in regard to prolonged standing, repetitive tasks, and exercise recommendations. Adjusting organizational measures, promoting and practicing frequent rest breaks, regular exercising, avoiding prolonged standing, and the formation of repetitive tasks delivering ongoing safety training is among the most potent essential measures required in preventing low back pain. The organization should implement and follow occupational health and safety service protocols.

### Strength and limitations of the study

This study assessed the burden of LBP among wait staff with a large sample size. Despite this, this study has certain limitations. The cross-sectional form of this study precludes a follow-up, which would have provided a better design for discovering variables connected to low back pain. Patients' self-reported data were also used to attain the results. This could have been influenced by recollection bias. Another possible limitation could be the absence of the control group, which makes it difficult to identify the actual proportion of low back pain resulting from the work condition.

## Data availability statement

The original contributions presented in the study are included in the article/Supplementary material, further inquiries can be directed to the corresponding author.

## Ethics statement

The studies involving human participants were reviewed and approved by university of Gondar Ethical Committee board. The patients/participants provided their written informed consent to participate in this study.

## Author contributions

MG, ESY, and KSA devised the initial concept, assisted in the writing of the proposal, created the study, and were involved at every stage of the project's execution. ESY and MG handled the data analysis, the first draft of the manuscript, and in charge of data analysis. ESY had double-checked, reran the data analysis, and considerably reworked the content before submission. KSA, ESY, MG, and AK conceptualized the basic idea, wrote the proposal, and critically revised the manuscript for essential intellectual content. All authors contributed to the article and approved the submitted version.

## Conflict of interest

The authors declare that the research was conducted in the absence of any commercial or financial relationships that could be construed as a potential conflict of interest.

## Publisher's note

All claims expressed in this article are solely those of the authors and do not necessarily represent those of their affiliated organizations, or those of the publisher, the editors and the reviewers. Any product that may be evaluated in this article, or claim that may be made by its manufacturer, is not guaranteed or endorsed by the publisher.
