# Supplementary material for: Low back pain and its determinants among wait staff in Gondar town, North West Ethiopia: A cross-sectional study
Source: Front Pain Res (Lausanne). 2022 Sep 6;3:964297. doi: 10.3389/fpain.2022.964297 (PMC9485721; doi:10.3389/fpain.2022.964297)
Supplement: Supplementary file 1 [file Data_Sheet_1.docx]

**Part I. Socio-demographic characteristics**

**Direction: Choose one possible answer and circle it.**

| **NO.** | **Question** | **Possible (alternative) Response** | **Remark** |
| --- | --- | --- | --- |
| 1.1 | Sex | 1. Female 2. Male |  |
| 1.2 | Age | year |  |
| 1.3 | Religion | 1. Orthodox   2. Catholic  3. Protestant  4. Muslim  5. Others specify |  |
| 1.4 | Educational level | 1. Can’t read & write 2. Can read and write 3. Primary school (1-8) 4. Secondary school (9-12) 5. Graduated from Technical and Vocational school |  |
| 1.5 | Marital status: | 1. Married 2. Single 3. Divorced 4. Widowed 5. Separated |  |
| 1.6 | DO you have additional part time job | 1. Yes 2. No |  |
| 1.7 | How is your work condition status? | 1. Day 2. Sometimes day sometimes night on shift 3. night |  |
| 1.8 | Year of experience | years |  |

**Part II. Personal factors**

**Direction: write one possible answer or circle it**

| **ID. No** | **Question** | **Answer** | **Remark** |
| --- | --- | --- | --- |
| 2.1 | What is your height? | ---------------meter |  |
| 2.2 | What is your weight? | ---------------- kg |  |
| 2.3 | What is your BMI? | ----------------kg/m2 |  |
| 2.4 | Have you taken special Ergonomic training on this work? | 1. Yes 2. No |  |
| 2.5 | Do you have Knowledge on Back Ergonomics? | 1. Yes 2. No |  |
| 2.6 | Do you have a habit of doing regular exercise? | 1. Never exercise 2. Sometimes 3. Usually |  |

**Part III. Psychological Factors**

**Direction: Choose one possible answer and circle it**

| ID. NO | Questions | Answer | Remark |
| --- | --- | --- | --- |
| 3.1 | Are you satisfied for being waiter? | 1. Yes 2. No |  |
| 3.2 | Are you comfortable with your daily activities in the hotel? | 1. Yes 2. No |  |
| 3.3 | Are you Mental stressed due to you have been working as a waiter? | 1. Yes 2. No |  |
| 3.4 | Do you have Sleep disturbance due to your work? | 1. Yes 2. No |  |
| 3.5 | Are you Bothered by feeling senseless and Little pleasure  due to your work ? | 1. Yes 2. No |  |
| 3.6 | You feel fatigue because of daily workload during your  work? | 1. Yes 2. No |  |
| 3.7 | Are you satisfied with the income you get from your work? | 1. Yes 2. No |  |
| 3.8 | Generally how much you are satisfied with your work? | 1. Very dissatisfied 2. Dissatisfied 3. Neutral Satisfied 4. Very satisfied |  |

**Part VI Low Back Pain (LBP) status**

**Direction: Choose one possible answer and If you answered NO to Question 4.1 do not answer The remaining questions**

| ID. NO | Questions | Answers | Remark |
| --- | --- | --- | --- |
| 4.1 | Have you had low back pain in the last 12 months since you were working as a waiter? | 1. Yes 2. No |  |
| 4.2 | Have you ever been hospitalized because of low back pain in  the last 12 months | 1. Yes 2. No |  |
| 4.3 | Have you ever been diagnosed MSDs by physician? | 1. Yes 2. No |  |
| 4.4 | If you answered yes to Question no 4.1 Please show us where you feel pain in the diagram by mark it. | 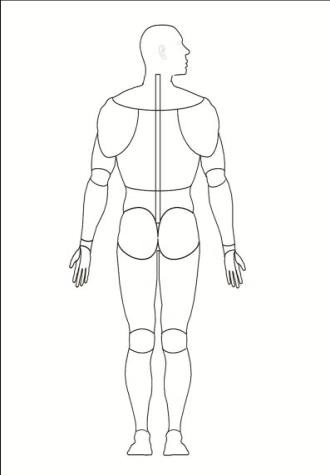 |  |
| 4.5 | Have you had low back pain during the last 7 days? | 1. Yes 2. No |  |
| 4.6 | What best describes the intensity of your LBP? | 1. Mild 2. Moderate 3. Severe |  |
| 4.7 | Do you feel Pain to Lower Extremities starting from lower back? | 1. Yes 2. No |  |
| 4.8 | Has the LBP affected you outside of work in terms of activities of daily living and leisure activities? | 1. Yes 2. No |  |
| 4.9 | Have you ever been absent from work in the past 6 month due to your low back pain? | 1. Yes 2. No |  |
| 4.10 | Have you ever thought to change your job because of low back pain? | 1. Yes 2. No |  |

**Part V Occupational and ergonomic Factors**

**Direction: Choose one possible answer but for question 5.2 you can choose (circle) more than one factor**.

| ID NO | Questions | Answers | Remark |
| --- | --- | --- | --- |
| 5.1 | Was your LBP start’s after started working as a waiter? | 1. Yes 2. No |  |
| 5.2 | Which work activities are the cause for your low back pain/ or aggravated your low back pain? (You can choose (circle) more than one factor). | 1. Bending / Twisting Lifting 2. Standing Sitting 3. Forming repetitive tasks 4. Working in an awkward / cramped position 5. Working when physically fatigued 6. Other: Please specify: |  |
| 5.3 | Did you feel pain on your low back more at night shift different from the day shift relatively? | 1. Yes 2. No |  |
